# The Therapeutic Crossroad Between Mitochondria and Cannabidiol: A Mini-Review

**DOI:** 10.3390/biology15060510

**Published:** 2026-03-22

**Authors:** Mihaela Jorgovan, Tamara Maksimović, Oana Bătrîna, Codruța Șoica, Alexandra Mioc, Marius Mioc

**Affiliations:** 1 Faculty of Pharmacy, “Victor Babes” University of Medicine and Pharmacy, Eftimie Murgu Square, No. 2, 300041 Timisoara, Romania; mihaela.coban@umft.ro (M.J.); oana.esanu@umft.ro (O.B.); codrutasoica@umft.ro (C.Ș.); alexandra.mioc@umft.ro (A.M.); marius.mioc@umft.ro (M.M.); 2 Research Centre for Experimental Pharmacology and Drug Design (X-Pharm Design), “Victor Babes” University of Medicine and Pharmacy, Eftimie Murgu Square, No. 2, 300041 Timisoara, Romania

**Keywords:** cannabidiol, mitochondria, cancer, antioxidant activity, nanoformulations

## Abstract

Cannabidiol is a non-psychoactive compound originating from *Cannabis sativa* L., with a promising therapeutic profile that influences numerous cellular processes. A major area of interest is its impact on mitochondria, organelles essential for cellular metabolism, ATP production, calcium homeostasis, and stress response. This article explores the available data on contribution of CBD effect on mitochondria to its therapeutic potential in treatment of various pathologies: cancer, cardiovascular, lung, neurological, gastrointestinal and liver disease, and muscle pathologies. Regarding cancer, the cytotoxic effects of cannabidiol on glioma, leukaemia, non-Hodgkin lymphoma, prostate, gastric, and breast cancer are analysed. In the case of cardiomyopathies and heart failure, cannabidiol plays an important role in reducing oxidative stress and promoting mitochondrial biogenesis. In lung diseases, cannabidiol reduces the expression of mitochondrial fission genes and increases the expression of fusion genes. When it comes to neurological pathologies, cannabidiol protects neurons and exhibits a strong antioxidant effect, while in gastrointestinal and liver diseases, cannabidiol stabilises mitochondrial membrane potential, increases ATP production, and reduces oxidative stress. In muscle affections, cannabidiol improves mitochondrial function by inhibiting excessive mitophagy. Although modern formulations may improve the low bioavailability of CBD, its potential non-selective cytotoxicity toward non-malignant cells remains an important concern that warrants further investigation. Nevertheless, cannabidiol possesses a remarkable therapeutic potential, and its effects on mitochondria open new perspectives in the treatment of numerous diseases.

## Simple Summary

Cannabidiol is a compound from the plant *Cannabis sativa* that does not produce psychoactive effects and has increasingly been studied for its therapeutic potential. This article addresses the role of cannabidiol in mitochondrial function, as mitochondria are essential for cellular energy transformation and for maintaining normal cellular responses to stress. The article reviews current research on the effects of cannabidiol on mitochondria in different disease models, including cancer, cardiovascular, pulmonary, neurological, gastrointestinal, liver, and muscle diseases. Although clinical application of cannabidiol is limited by its low systemic availability and non-selective cytotoxicity, recent advances in formulation may help overcome this limitation. Overall, the effects of cannabidiol on mitochondrial function indicate its potential relevance as a therapeutic approach for a wide range of diseases and identifies directions for future research.

## 1. Introduction

Cannabidiol (CBD) is a lipophilic compound that belongs to the phytocannabinoid class, characterised by the presence of a C21 terpeno-phenolic skeleton in its structure [1,2]. It is one of the 554 compounds that can be found in *Cannabis sativa* (marijuana, hemp) [2] and represents the main non-psychoactive substance derived from this plant, since it does not possess any psychotropic activity. However, CBD can influence numerous processes, such as cell survival, inflammation, and oxidative damage [2] and, on a molecular level, it can bind to around 56 targets from different types of channels and receptors to various enzymes [3]. In recent years, CBD has become a point of interest for many researchers due to its therapeutic potential [4], showing effects in the treatment of chronic pain, anxiety, cancer, cardiovascular and psychiatric pathologies [2], epilepsy (approved by the FDA), and multiple sclerosis (approved in the UK) [5]. CBD is rarely associated with serious side effects and it is considered a safe drug [2].

Mitochondria are organelles that have a role in ATP production, ROS generation, fatty acid beta-oxidation, and Ca^2+^ homeostasis, therefore influencing cellular metabolism, homeostasis maintenance, and stress responses [6]. It is due to this crucial role in cellular functions that mitochondria also participate in the pathogenesis of inflammation and various diseases, such as diabetes, cardiovascular and neurodegenerative disorders, cancer, and inflammation [7]. More precisely, diseases can be correlated with imbalanced mitochondrial dynamics and mitochondrial dysfunction [6]. Hence, the mitochondria represent a promising target for pharmacological interventions with the aim of treating numerous diseases [7]. Studies have shown that CBD has the ability to influence mitochondrial processes and functions, such as mitochondrial biogenesis, fusion, fission, respiration, cell death, mitochondrial DNA epigenetics (DNA hydroxymethylation), and monoamine oxidase (MAO) activity. However, many aspects of its effects remain unclear [8].

Considering that mitochondria have an important role in the pathogenesis of numerous diseases, by modifying mitochondrial functions, CBD could represent a new therapeutic option for the treatment of chronic and degenerative diseases. This study aims to investigate the effects of CBD in various diseases, focusing on its ability to influence mitochondrial functions.

**Figure 1:**
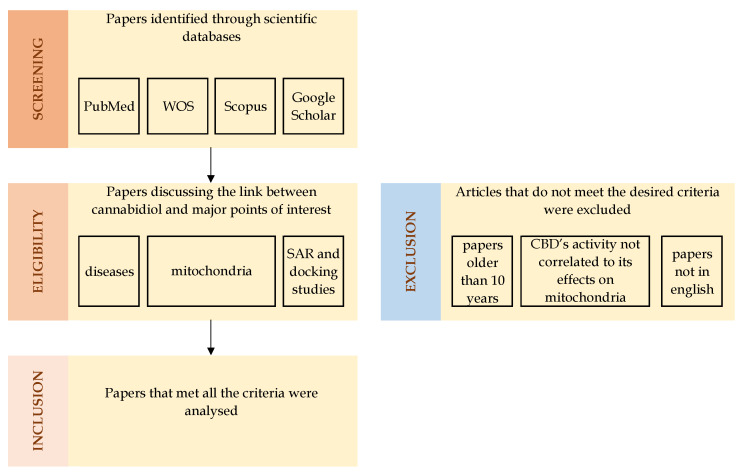
Flow diagram describing the data selection process: the first phase consisted of screening, in which scientific databases such as PubMed, Web of Science, Scopus, and Google Scholar were used to identify papers of interest; the second phase involved the establishment of eligibility criteria: papers discussing the link between cannabidiol and disease models, mitochondria and/or structure–activity relationship were selected for furhter analysis, while papers older than 10 years, papers where CBD’s effects were not mitochondria-mediated, and articles not in English were excluded; finally, the last phase included the analysis of the selected papers; WOS- Web of Science, SAR-structure–activity relationship.

The current paper analysed the recent (last 10 years) articles regarding cannabidiol’s effects in models of various diseases, while exploring the link between its activity and mitochondrial function. We used several scientific databases, i.e., PubMed, Scopus, Google Scholar and Web of Science, searching for both in vitro and in vivo studies that discuss cannabidiol, pathologies, and mitochondria. Moreover, we reviewed and discussed studies addressing structure–activity relationships of CBD. We excluded articles where CBD’s activity could not be attributed to its effects on mitochondria (Figure 1).

## 2. Cannabidiol: Structure, Receptor Interactions, and Mitochondrial Effects

### 2.1. CBD Structure and Structure–Activity Relationship

**Figure 2:**
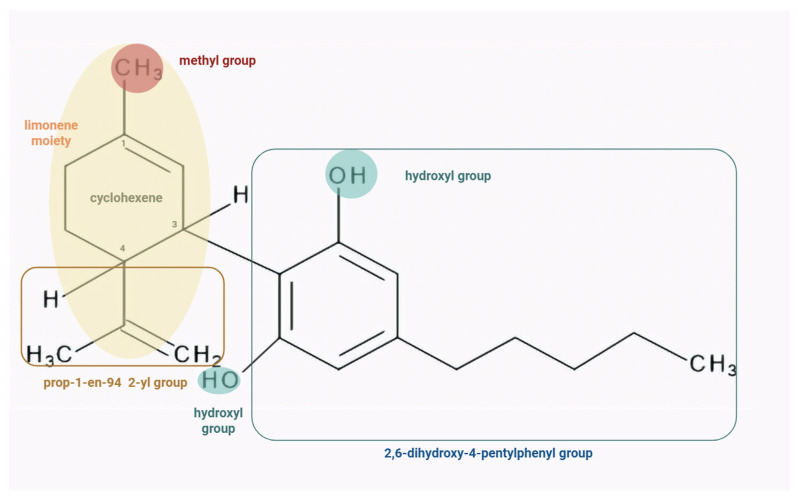
The chemical structure of CBD consists of a cyclohexene ring substituted with a methyl group at C1 (red circle), a 2,6-dihydroxy-4-pentylphenyl moiety at C3 (blue rectangle), and a prop-1-en-2-yl group at C4 (brown rectangle). Key functional groups involved in its activity, including the phenolic hydroxyl groups (blue circle) and the limonene-derived moiety (orange circle) are highlighted. Shorter alkyl side chains attached to the phenolic ring may decrease the CB1/CB2 binding affinity, whereas longer chains (e.g., hexyl, heptyl and octyl) could enhance it. Etherification or removal of the phenolic hydroxyl group could lead to an increase in CB2 selectivity. Moreover, shorter side chains may reduce CB receptor activity, whereas longer chains may increase pharmacological potency. Also, modifications of the phenolic hydroxyl group could markedly alter activity [12].

Regarding its molecular structure, CBD is a cyclohexene substituted by a methyl group at position 1, a 2,6-dihydroxy-4-pentylphenyl group at position 3, and a prop-1-en-2-yl group at position 4 (Figure 2). Its molecular formula is C_21_H_30_O_2_, with a molecular weight of 314.5 g/mol [9]. Although structurally similar to psychoactive tetrahydrocannabinol (THC), CBD contains one more hydroxyl group and lacks a cyclic ring, thus having different pharmacological properties [2]. Owing to the presence of a large hydrophobic region, CBD exhibits high lipophilicity and low hydrophilicity [10]. Due to these properties and its small size, CBD can penetrate highly vascularised tissues and cross plasma membranes, entering the cytosol [2,11].

By definition, chemical substances known as cannabinoids have the ability to interact with cannabinoid receptors due to their structure [2].

Structure–activity relationship (SAR) studies focusing on cannabinoid receptors (CB1 and CB2) indicate that receptor binding is primarily determined by the hydrophobic alkyl side chain and the phenolic hydroxyl groups (Figure 2) [12].

### 2.2. CBD-Mediated Mitochondrial Modulation

Cannabinoids’ effects on mitochondria were initially explained by the direct binding of the compounds to the mitochondrial membrane, leading to structural and functional alterations [13].

**Figure 3:**
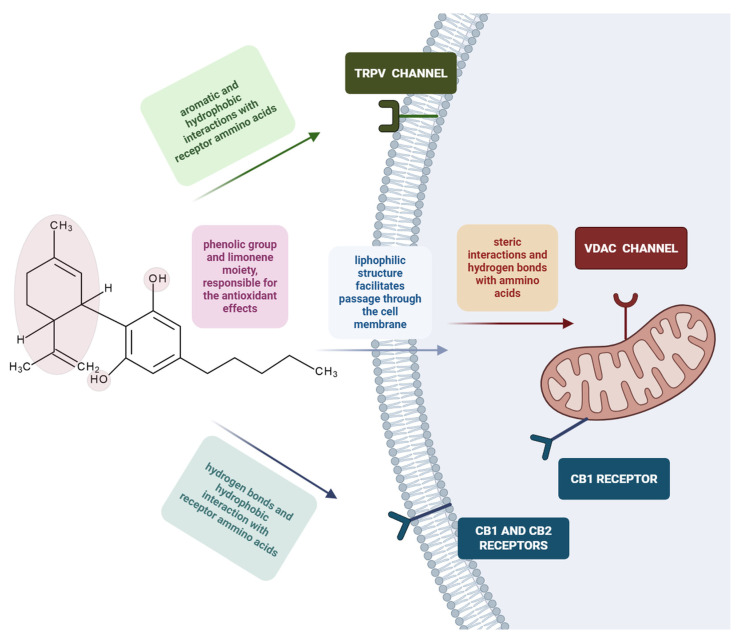
Interactions between CBD and different cellular components, based on the structure–activity relationship. CBD exerts its antioxidant activity through its phenolic hydroxyl groups and limonene moiety; it forms aromatic and hydrophobic interactions with amino acids present in the structure of the TRPV channel, localised in the cell membrane; it forms hydrogen bonds and hydrophobic interactions with CB receptor amino acids, localised both in the cell membrane (CB1 and CB2) and outer mitochondrial membrane (CB1). Due to its lipophilic structure, CBD can pass through the cell membrane and can interact with intracellular channels and receptors. It forms hydrogen bonds and steric interactions with amino acids present in the VDAC channel, localised in the outer mitochondrial membrane. The figure was created with the help of BioRender.com.

Later studies suggested that cannabinoids have the ability to modulate mitochondrial activity due to cannabinoid receptor activation, localised in the cell membrane and in the outer mitochondrial membrane (Figure 3) [13]. Newer findings have shown that CBD acts both as an antagonist (CB1 receptor) and a partial agonist (CB2 receptor), depending on the cannabinoid receptor subtype, CB1 or CB2 [14], while mitochondria-mediated cytotoxic effects are associated only with CB1 subtype, along with other types of receptors (Table 1). However, CBD shows low affinity for both CB1 and CB2 receptors (Table 1) [8]. At the same time, as CBD interacts with several non-cannabinoid receptors, such as serotonin receptor 1A, vanilloid receptor 1, adenosine A2A receptor, and peroxisome proliferator-activated receptor gamma Mitochondrial functions are influenced by several pathways and processes [8].

CBD has also been shown to exhibit antioxidant properties not by interacting with any receptors or enzymes but simply due to the presence of two hydroxyl groups in its chemical structure [10]. Briefly, the antioxidant effect is primarily exerted through the abstraction of a hydrogen atom and an electron from the phenyl group of the antioxidant molecule. This process involves the free radical removing of a hydrogen atom from the antioxidant, while the antioxidant simultaneously donates an electron to the free radical, thereby neutralising it and exerting its protective effect. Furthermore, the antioxidant capacity is also linked to the limonene moiety present in the CBD structure [15]. The antioxidant capacity of CBD was superior to that of tocopherol and ascorbate when tested in neurons [16].

In addition to its well-documented antioxidant properties, CBD has also been reported to exert pro-oxidant effects in a dose-, time-, and cell type-dependent manner, inducing mitochondrial ROS production and oxidative stress in various cellular models [17].

**Figure 4:**
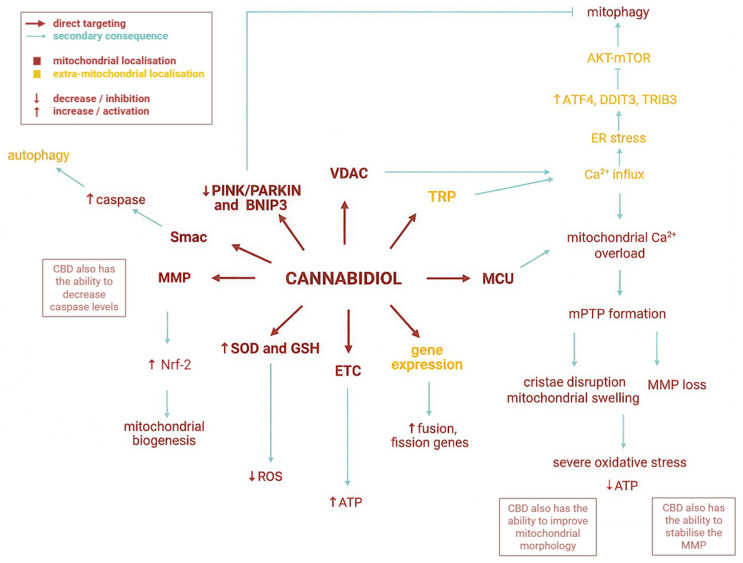
CBD’s biological effects [24,25,26,27,28,29,30,31]. It can be observed that CBD exerts its activity through both direct mitochondrial targeting (CBD binds to VDAC, influences the electron transport chain complexes, activates mitochondrial stress responses, etc., leading to different mitochondria-associated effects) and indirect mitochondrial consequences (CBD initially interacts with TRP, increases Ca^2+^ influx, modulates endoplasmic reticulum activity, etc., which ultimately leads to changes in mitochondrial processes, without the direct interaction between the compound and mitochondrial structures). Cannabidiol can also exhibit bidirectional effects (ROS neutralisation and generation, ATP production etc.), depending on the pathological context. ATP—adenosine triphosphate. ER—endoplasmatic reticulum; ETC—electron transport chain; GSH—glutathione; MCU—mitochondrial calcium uniporter; MMP—mitochondrial membrane potential; mPTP—mitochondrial permeability transition pore; SOD—superoxide dismutase. The figure was created with the help of BioRender.com.

Concerning the mechanisms underlying mitochondrial process modulation, CBD can influence mitochondrial function both directly and indirectly, as represented in Figure 4. The direct mitochondrial targeting includes direct interaction with mitochondrial receptors and signalling pathways, while indirect targeting is achieved through upstream signalling.

**Table 1:** CBD-receptor interactions resulting in mitochondrial effects and cytotoxic activity. TRPV—Transient receptor potential Vaniloid receptor channels, VDAC—Voltage-dependent anion channel, CB—cannabinoid receptor, Phe—Phenylalanine, Val—Valine, Asn—Asparagine, Tyr—tyrosine, Thr—Threonine, Gly—Glycine, His—Histidine, Leu—Leucine, Gln—Glutamine, Ala—Alanine, Asp—Aspartic acid, Ile—Isoleucine, ROS—reactive oxygen species, mPTP—mitochondrial permeability transition pore, MMP—mitochondrial membrane potential, NCX—sodium-calcium exchanger, ER—endoplasmic reticulum, MCU—mitochondrial calcium uniporter.

Receptor Type	Receptor Subtype	Localisation	Binding	Binding Affinity	Effect	Effects on Mitochondria Due to CBD-Receptor Binding	Reference
Transient receptor potential Vaniloid receptor (TRPV) channels	TRPV1	cell membrane (nervous system, epithelium).	Chain A of the receptor, (Phe, Val and Asn ammino acids) and chain B (Phe), through aromatic and hydrophobic interactions.	strong	agonist	CBD → TRPV1 activation → Ca^2+^ imbalance → ROS production → impaired MMP	[18]
	TRPV2	cell membrane (nervous system).	Hydrophobic pocket of the receptor (aromatic and hydrophobic residues in the S5 and S6 helices), through pi-stacking and hydrophobic interactions.	strong	agonist	CBD → TRPV2 activation → increase in intracellular Ca^2+^ levels → potential mitochondrial Ca^2+^-dependent effects. No direct evidence of mitochondrial effects via TRPV2. Mitochondrial Ca^2+^-dependent effects reported for CBD include altered mitochondrial Ca^2+^ homeostasis, modulation of mitochondrial Ca^2+^ transporters (NCX: alters Ca^2+^ flux depending on cell state; VDAC1: reduces channel conductance, promoting Ca^2+^ accumulation and apoptosis), bidirectional regulation of Ca^2+^ signalling (increasing basal Ca^2+^ in resting cells, reducing oscillations in highly excitable cells), and, under higher Ca^2+^ loads, mPTP and mitochondrial-driven apoptosis.	[8,18,19]
	TRPV4	cell membrane (muscle tissue, epithelial tissue).	Chain A of the receptor (Tyr, Asn, Phe), through hydrophobic interactions.	strong	agonist	CBD → TRPV4 activation → increase in intracellular Ca^2+^ levels → mitophagy initiation through ER stress and the ATF4–DDIT3–TRIB3–AKT–mTOR axis, and other potential mitochondrial Ca^2+^-dependent effects. Mitochondrial Ca^2+^-dependent effects reported for CBD include altered mitochondrial Ca^2+^ homeostasis, modulation of mitochondrial Ca^2+^ transporters (NCX: alters Ca^2+^ flux depending on cell state; VDAC1: reduces channel conductance, promoting Ca^2+^ accumulation and apoptosis), bidirectional regulation of Ca^2+.^ signalling (increasing basal Ca^2+^ in resting cells, reducing oscillations in highly excitable cells), and, under higher Ca^2+^ loads, mPTP and mitochondrial-driven apoptosis.	[8,18,20]
Voltage-dependent anion channel (VDAC)	VDAC1	outer mitochondrial membrane	CBD phenolic groups form steric interactions with Thr and Gly, and hydrogen bonds with Gln, His, and Leu.	strong	Stabilises VDAC1 in a Ca^2+^-permeable subconductance state	CBD → VDAC1 → increase in Ca^2+^ permeability → Entrance of Ca^2+^ into the intermembrane space → MCU-mediated mitochondrial matrix Ca^2+^ overload → mitochondrial permeability transition pore formation, mitochondrial membrane potential loss, cristae disruption, mitochondrial swelling → dysfunction of mitochondria, loss of ATP production, severe oxidative stress.	[11,18]
Cannabinoid receptors (CB)	CB1	cell membrane, outer mitochondrial membrane	CBD hydroxyl group forms a hydrogen bond with Asp, while its alkyl chain forms hydrophobic contacts with Phe, Ile, Ile and Phe.	strong (predicted), low (experimental).	antagonist	CBD may indirectly modulate mitochondrial processes by antagonising CB1 receptor signalling, as CB1 activation has been shown to suppress mitochondrial respiration and energy metabolism; however, several mitochondrial effects of CBD have also been reported to occur independently of CB1.	[14,18,21,22]
	CB2	cell membrane	CBD hydroxyl group forms H-bond with Val and hydrophobic interactions with Phe, Ala and Trp.	strong (predicted), low (experimental).	partial agonist	No direct CB2-mediated mitochondrial effects of CBD have been identified in the available literature.	[23]

## 3. CBD and Mitochondria in Disease

### 3.1. CBD and Mitochondria in Cancer

**Figure 5:**
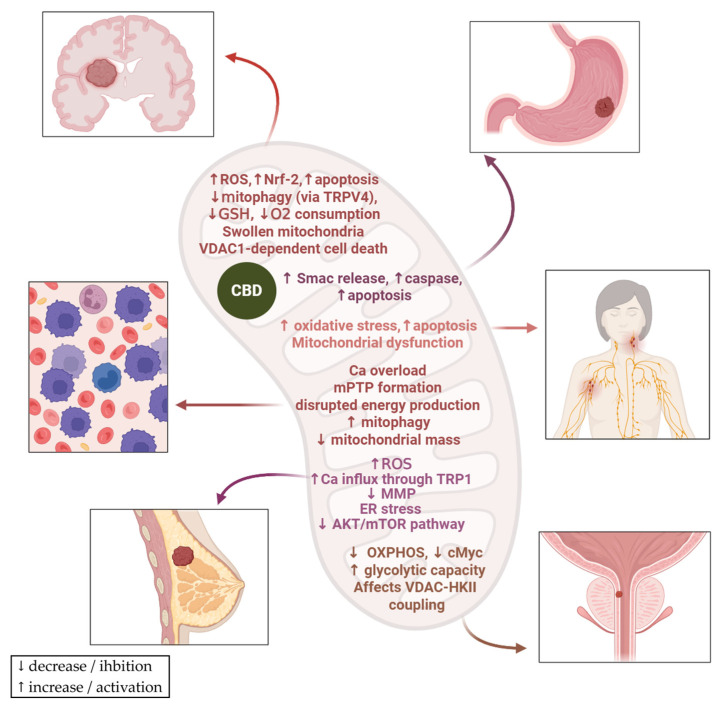
CBD’s effects on mitochondria in different types of cancer (glioma, gastric cancer, leukaemia, lymphoma, breast and prostate cancer). In glioma models, CBD increases ROS and Nrf-2 levels, stimulates apoptosis, decreases mitophagy through TRPV4, reduces glutathione levels, decreases oxygen consumption, leads to mitochondrial swelling and VDAC1-dependent cell death. In gastric cancer models, CBD increases Smac release and caspase levels and stimulates apoptosis. In the lymphoma model, it increases oxidative stress, stimulates apoptosis and leads to mitochondrial dysfunction. In leukaemia models, CBD induces Ca overload, leads to mitochondrial permeability transition pore formation and disrupted energy production, increases mitophagy and reduces mitochondrial mass. In breast cancer models, CBD increases ROS and calcium influx through TRPV1, reduces mitochondrial membrane potential, induces endoplasmic reticulum stress and inhibits AKT/mTOR pathway. In prostate cancer models, CBD decreases oxidative phosphorylation, reduces cMyc, increases glycolytic capacity and affects VDAC–HKII coupling. ER—endoplasmatic reticulum; GSH—glutathione; mPTP—mitochondrial permeability transition pore; MMP—mitochondrial membrane potential; OXPHOS—oxidative phosphorylation; HK—hexokinase. The figure was created with the help of BioRender.com.

Cancer, the second leading cause of death worldwide, is a pathology characterised by the uncontrolled proliferation of cells [32]. Despite numerous treatments, many patients do not achieve long-term survival [32]. Mitochondrial dysfunction, caused by abnormalities such as defects in the tricarboxylic acid cycle enzymes, mutations in mitochondrial DNA, impaired electron transport chain, oxidative stress, or altered oncogene and tumour suppressor signalling, has been identified across various types of human cancers [33]. Therefore, these mitochondrial signalling pathways are emerging as important targets for new anticancer agent development. In this context, mitocans are compounds that target mitochondria in cancer cells to cause growth inhibition or apoptosis [34]. Evidence from multiple studies demonstrates that a large number of various phytochemicals are able to decrease cell viability by inducing mitochondrial dysfunction in cancer cells [35,36]. Glioma remains among the most aggressive and deadly forms of cancer, despite recent progress in therapeutic approaches [37]. Massi et al. demonstrate that CBD selectively induces apoptosis in glioma cells by activating the caspase cascade, increasing ROS and depleting glutathione. These effects are not seen in normal glial cells, highlighting its potential as a targeted antitumor agent [38]. Another study by Huang et al. showed that CBD induces autophagic cell death in human glioma cells through mitochondrial dysfunction and lethal mitophagy arrest. These effects represent a secondary consequence of the calcium flux through the TRPV4 receptor, which plays a key role in mitophagy initiation by activating endoplasmic reticulum stress and the ATF4–DDIT3–TRIB3–AKT–mTOR axis. TRPV4 expression was also found to correlate with tumour grade and poor survival in glioma patients [25]. Gross et al. investigated the in vitro cytotoxic effect of CBD on human and canine glioma cells, and their results showed that CBD reduces cell viability in a dose-dependent manner. This cytotoxic effect is linked to mitochondrial dysfunction, characterised by swollen mitochondria and reduced oxygen consumption [39]. Notably, when the mitochondrial channel VDAC1 was inhibited, cell death was prevented, indicating that CBD kills glioma cells by disrupting calcium homeostasis and mitochondrial function, likely through a VDAC1-dependent mechanism [39]. Supporting this mechanism, a separate study in BV-2 microglial cells demonstrated a direct interaction between CBD and the outer mitochondrial membrane protein VDAC1, resulting in reduced channel conductance and mitochondrial dysfunction leading to cell death [40]. Glioblastoma multiforme (GBM) is the most aggressive and widespread form of glioma, and it is the most common primary brain tumour in adults [41]. Giannotti et al. investigated the effects of CBD on U87MG glioblastoma cells. Results showed that CBD reduced cell viability by altering mitochondrial membrane potential and redox status. It triggered mitochondrial stress responses, including increased nuclear localisation of the Nrf-2 involved in mitochondrial biogenesis, and enhanced autophagic flux (Figure 5). However, despite the induction of pro-apoptotic proteins, apoptosis was not fully activated [42]. Also, other studies mention a CBD-caused inhibition of pro-oncogenic Nrf-2, favourable for cancer suppression [43]. Overall, CBD disrupted mitochondrial function and dynamics, making glioma cells more vulnerable to cytotoxic effects, thus exhibiting therapeutic potential through the modulation of mitochondrial processes and autophagy pathways [42]. A study by Rupprecht et al. investigated the anticancer effects of the combination of CBD and THC (tetrahydrocannabinol) on glioblastoma cells. The results showed that this treatment reduces the oxygen consumption rate, a well-known measure of mitochondrial function. This decrease is associated with a reduction in the levels of proteins belonging to the electron transport chain complexes, primarily complexes I and IV [44].

Leukaemia is a diverse set of blood cancers that develop due to the abnormal growth of immature white blood cells [45]. Acute lymphoblastic leukaemia is characterised by the cancerous transformation and rapid growth of immature lymphoid cells in the bone marrow, bloodstream, and other tissues outside the marrow. Although it primarily affects children, it is particularly severe and challenging when diagnosed in adults [46]. Olivas-Aguirre et al. demonstrated in their study that CBD has cytotoxic effects in acute lymphoblastic leukaemia (T-ALL) by targeting mitochondria. It induces mitochondrial calcium overload, leading to the formation of a stable mitochondrial permeability transition pore (mPTP) and cell death. This mitochondrial disruption is a key mechanism by which CBD kills leukaemia cells, especially when combined with drugs like tamoxifen that sensitise cells by affecting mitochondrial calcium regulation [47]. Chronic myeloid leukaemia is a blood disorder caused by a genetic change in stem cells, leading to the overproduction of granulocytes and resulting in spleen enlargement and high white blood cell levels [48]. Maggi et al. studied the effect of CBD in chronic myeloid leukaemia cells and reported anticancer effects based on mitochondrial-related mechanisms that include: (1) induction of mitochondrial dysfunction by impairing mitochondrial function, disrupting energy production and cellular metabolism; (2) indirect activation of mitophagy, which selectively degrades damaged mitochondria and promotes cell death; and (3) a reduction in mitochondrial mass, reflecting the elimination of mitochondria through mitophagy (Figure 5) [49].

Mahmoud et al. demonstrated that CBD has anticancer effects on hormone-refractory prostate cancer (HRPC) through mitochondria-related mechanisms, as follows: (1) it modulates metabolic plasticity by affecting the coupling between Voltage-Dependent Anion Channel 1 (VDAC1) and hexokinase II (HKII) on the outer mitochondrial membrane, which alters essential mitochondrial functions required for cancer cell survival; (2) it inhibits oxidative phosphorylation and reduces mitochondrial energy transformation; and (3) it increases glycolytic capacity, leading to an energetic imbalance between the Warbourg effect and oxidative phosphorylation. Thus, CBD targets mitochondria by disturbing energy metabolism and blocking oncogenic signalling pathways, such as cMyc, contributing to the death of HRPC cells (Figure 5) [50].

Jeong et al. showed that CBD induces apoptosis in gastric cancer cells by causing mitochondrial dysfunction, leading to the modulation of the Smac/XIAP pathway (Figure 5). Specifically, CBD promotes the release of Smac from mitochondria, which inhibits XIAP and activates caspases, ultimately leading to cancer cell death. This mechanism suggests that targeting Smac/XIAP with CBD could offer therapeutic potential against gastric cancer [26].

Breast cancer is among the most common types of cancer and poses significant challenges because of its heterogeneity and the development of resistance to treatments [51]. A study demonstrates that CBD induces breast cancer cell death through a complex mechanism involving both apoptosis and autophagy [27]. CBD causes endoplasmic reticulum stress, inhibits the AKT/mTOR pathway, activates the intrinsic apoptotic pathway via BID translocation and cytochrome c release, and indirectly reduces the mitochondrial membrane potential. Additionally, CBD increases ROS levels, which are essential for the induction of both autophagy and apoptosis (Figure 5) [27]. Another study indicated that CBD induces endoplasmic reticulum stress in breast cancer cells by increasing the calcium influx through the TRPV1 receptor, simultaneously increasing mitochondrial Ca^2+^ levels, leading to raised reactive oxygen species levels, which disrupts protein folding [52].

Non-Hodgkin lymphoma (NHL) is a malignancy of lymphoid tissues that originates from B-cell precursors, mature B cells, T-cell precursors, or mature T cells. There is a wide range of NHL subtypes, each with distinct characteristics and various responses to treatment [53]. Omer et al. studied the cytotoxic effects of three cannabinoids, including CBD, on canine B- and T-cell lymphoma lines as well as a human B-cell lymphoma line. The results showed that all three compounds decreased T-cell lymphoma viability and induced oxidative stress, inflammation, apoptosis, and mitochondrial dysfunction in the canine B-cell lymphoma (Figure 5) [54]. Overall, CBD was the most effective among the tested compounds [54].

Although it demonstrates cytotoxic activity in various cancer models, CBD may exert non-selective cytotoxic effects, potentially affecting non-malignant cell types such as microglia, oligodendrocytes, and other neural cells, which could limit its clinical applicability [55]. These non-selective cytotoxic effects appear to be dose- and time-dependent. For instance, Montes-de-Oca-Saucedo et al. observed that at concentrations exceeding 5 μM, the healthy cell lines HaCaT and HUVEC suffered significant morphological changes and loss of cell adhesion, with these effects becoming particularly potent during the 96 h of exposure [56].

### 3.2. CBD and Mitochondria in Rheumatoid Arthritis

**Figure 6:**
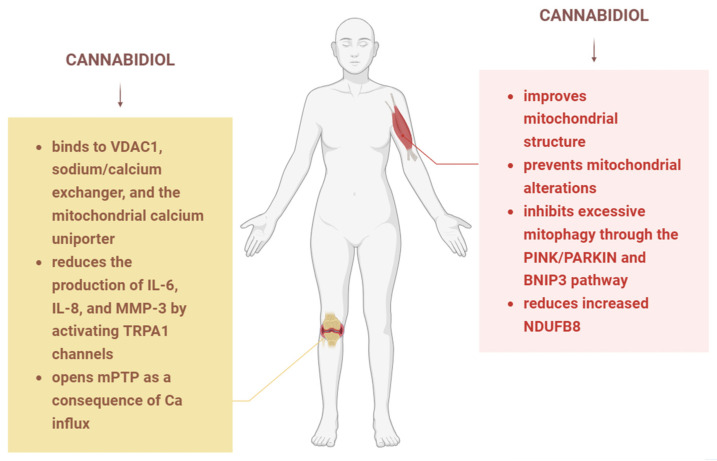
The effects of CBD in rheumatoid arthritis and myopathies. In rheumatoid arthritis models, CBD binds to the VDAC1 channel, the sodium–calcium exchanger and the mitochondrial calcium uniporter; it activates TRPA1 channels and consequently reduces the production of interleukins and MMP-3; it opens mPTP due to calcium influx. In miopathies, CBD prevents mitochondrial alterations and improves mitochondrial structure, inhibits excessive mitophagy and reduces the increased NDUFB8. IL—interleukin, MMP—matrix metalloproteinase, mPTP—mitochondrial permeability transition pore, TRPA1—transient receptor potential ankyrin, VDAC—Voltage-dependent anion channel. Image created with the help of BioRender.com.

Rheumatoid arthritis (RA), a widespread autoimmune disease, is characterised by chronic inflammation of multiple joints (synovitis) and progressive destruction of bone and cartilage [57]. A key mechanism underlying bone loss in RA is the activation of osteoclasts, a process driven by RANKL (Receptor Activator of NF-κB Ligand), a protein that promotes osteoclast formation and activity [57]. Synovial fibroblasts are the primary source of RANKL in the RA synovium and play a crucial role in both sustaining inflammation and mediating bone erosion [57]. A study showed that CBD has anti-arthritic effects and may ameliorate RA disease by targeting synovial fibroblasts in inflammatory conditions. Specifically, CBD increases intracellular calcium levels, decreases the viability of these cells, and reduces the production of IL-6, IL-8, and MMP-3 by activating transient receptor potential ankyrin (TRPA1), while opening the mitochondrial transition pore as a consequence (Figure 6). Moreover, it was observed that CBD binds preferentially to mitochondrial proteins, targeting VDAC1, the sodium/calcium exchanger, and the mitochondrial calcium uniporter (MCU). CBD’s effects were more pronounced when cells were pre-treated with TNF, indicating that CBD preferentially targets activated, pro-inflammatory rheumatoid arthritis synovial fibroblasts [58].

### 3.3. CBD and Mitochondria in Kidney Disease

Cisplatin is a frequently used chemotherapeutic drug in the treatment of various human cancers, being effective against different types, including carcinomas, germ cell tumours, sarcomas, and lymphomas. However, it has numerous side effects, one of the most severe being kidney damage [59]. Pan et al. studied the effect of CBD on cisplatin-induced nephrotoxicity in mouse models. Cisplatin caused an increase in ROS production, elevated nitric oxide synthase levels, stimulated the formation of nitrotyrosine, increased apoptosis and inflammation in mouse kidneys. These changes were associated with histopathological damage and impaired renal function [60]. CBD reduced the oxidative and nitrosative stress induced by cisplatin, decreased inflammation and cell death in the kidneys, and improved renal function. These findings suggest that CBD may represent a promising new approach as protection against cisplatin-induced nephrotoxicity [60].

**Figure 7:**
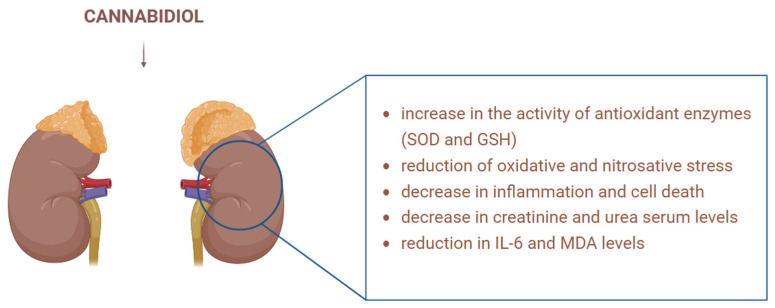
CBD’s beneficial effects in kidney pathology: It increases SOD and GSH levels, reduces oxidative and nitrosative stress, decreases interleukin, MDA, urea and creatinine levels, and reduces inflammation and cell death. SOD—superoxide dismutase; GSH—glutathione; IL—interleukin; MDA—malondialdehyde. Image created with the help of BioRender.com.

Doxorubicin is a chemotherapeutic agent used for the treatment of numerous types of solid tumours. Still, its clinical use is limited by its associated nephrotoxicity [61]. Soliman et al. studied the effect of CBD on doxorubicin-induced kidney dysfunction, tissue alterations, and inflammation in rat models. The results showed that CBD administration in rat models led to improved oxidative stress parameters (Figure 7). Specifically, it increased the activity of antioxidant enzymes (SOD and GSH), decreased serum levels of creatinine and urea, and reduced IL-6 and MDA levels, thus confirming its anti-inflammatory and antioxidant effects. Overall, the renal protective effects of CBD were highlighted and confirmed by histopathological analysis [62].

### 3.4. CBD and Mitochondria in Cardiomyopathies

**Figure 8:**
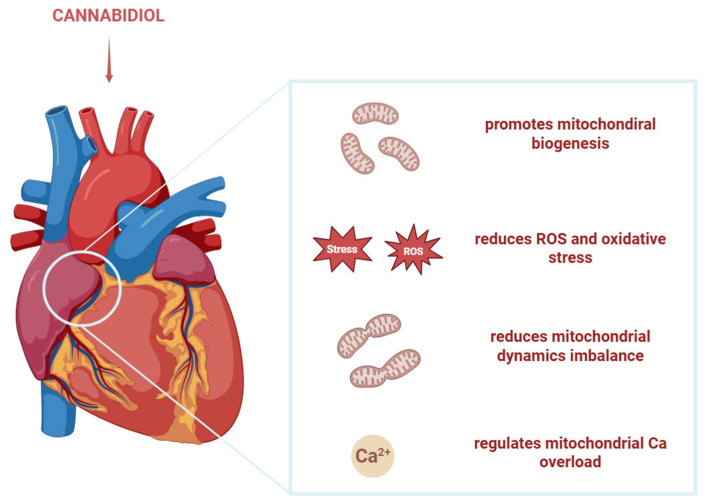
CBD’s effects on mitochondria in cardiomiopathies. CBD promotes mitochondrial biogenesis, reduces ROS, oxidative stress and mitochondrial dynamics imbalance and regulates calcium overload. The figure was created with the help of BioRender.com.

Cardiomyopathies are associated with a pro-inflammatory profile, alterations in oxidative phosphorylation and mitochondrial reactive oxygen species (ROS), as well as disturbances in the handling of intracellular Ca^2+^ [63]. Hao et al. studied the effect of CBD on doxorubicin-induced cardiomyopathy/heart failure in mouse models and revealed that CBD attenuated oxidative and nitrative stress, mitochondrial dysfunction, inflammation and cell death, and promoted mitochondrial biogenesis [64]. In another study, García-Rivas et al. evaluated the in vivo effects of CBD in a mouse model of induced heart failure. The results indicated that CBD helped maintain cellular oxidative status and mitochondrial bioenergetics, and regulated mitochondrial calcium overload through the modulation of MCU expression, which contributes to its cardioprotective effects (Figure 8) [29]. Wang et al. showed that CBD was able to diminish heart injury induced by perfluorooctanesulfonic acid in murine models by enhancing the antioxidant capacity, improving energy metabolism disorders that lead to cardiomyocyte death, and reducing mitochondrial dynamics imbalance [65].

### 3.5. CBD and Mitochondria in Pulmonary Disease

**Figure 9:**
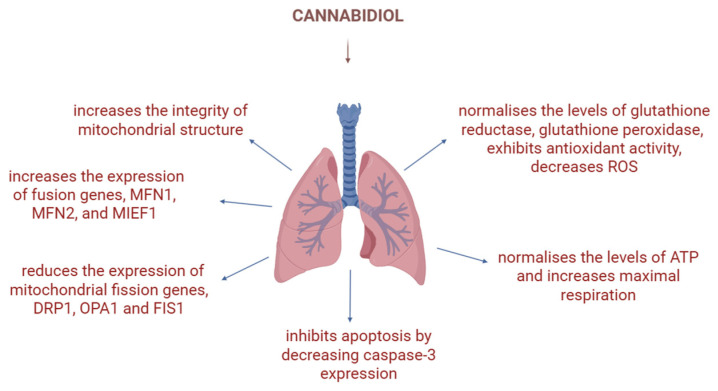
Various effects of CBD on mitochondria in pulmonary disease: CBD normalises the levels of antioxidant enzymes, decreases ROS, increases maximal respiration and normalises ATP levels, inhibits apoptosis, reduces the expression of fission genes and increases the expression of fusion genes, and increases the integrity of mitochondrial structure. Image created with the help of BioRender.com.

Pulmonary arterial hypertension (PAH) is a pathology characterised by increased proliferation of smooth muscle cells found in pulmonary arteries [28] and increased pulmonary vascular resistance [66]. It is associated with poor prognosis, and there are currently no drugs that can cure this condition [66]. PAH development is closely linked to mitochondrial dysfunction, where the Krebs cycle, ROS production, redox homeostasis, and mitophagy can all be affected [66]. It has been found that CBD, when tested on human and mouse pulmonary artery smooth muscle cells (hypoxia-induced/PAH), influences the altered mitochondrial activity by reducing the expression of mitochondrial fission genes, DRP1, OPA1, MIEF1 and FIS1, while increasing the expression of fusion genes, MFN1, MFN2, OPA1 and MIEF1 (Figure 9). Moreover, it increases the integrity of mitochondrial structure, normalises the levels of glutathione reductase, glutathione peroxidase, and ATP, increases maximal respiration, and decreases ROS. Ultimately, these effects alleviate the pulmonary arterial hypertension in a manner comparable to first-line drugs, bosentan and beraprost sodium [28].

In another study, conducted by Emre et al., CBD showed beneficial effects in the case of lung contusions caused by chest trauma; more specifically, CBD exhibited dose-dependent antioxidant activity, although the respective effect was more pronounced in the presence of inflammation. Moreover, CBD inhibits apoptosis by decreasing caspase-3 expression, thereby influencing the mitochondrial apoptosis pathway [67].

### 3.6. CBD and Mitochondria in Neuronal Pathologies

Existing evidence suggests that CBD possesses neuroprotective properties [5]; namely, CBD has shown protective effects against cerebral ischemia, a pathology linked to the modification of mitochondrial functions such as deficient bioenergetics and reduced redox conservation. When tested in oxygen–oxygen-glucose-deprivation/reperfusion mice model, CBD improved basal respiration, reduced ROS, and normalised antioxidant biomarkers [5].

**Figure 10:**
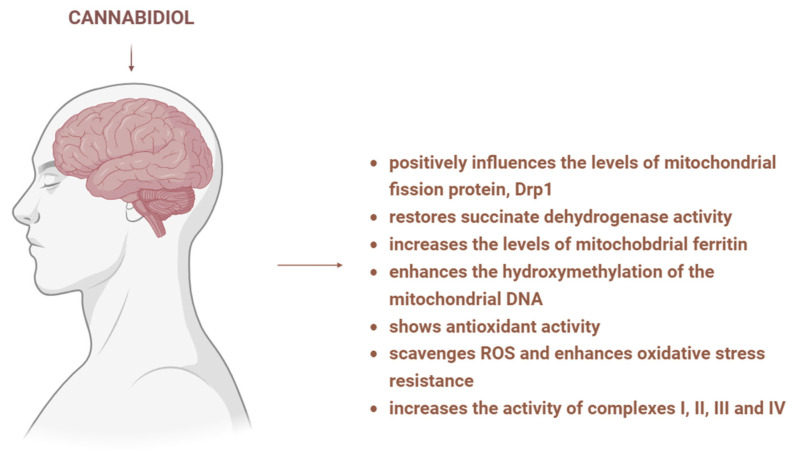
Mechanisms underlying CBD’s effects in neuronal pathology. CBD influences mitochondrial fission proteins, restores enzyme activity, increases mitochondrial ferritin level, induces epigenetic modifications, scavenges ROS and increases the activity of electron transport chain complexes. Image created with the help of BioRender.com.

Neurodegenerative pathologies are associated with the accumulation of transition metals, as can be seen in Parkinson’s, Alzheimer’s, and Huntington’s disease, leading to oxidative damage. It was reported that CBD positively influences iron-induced alterations, such as memory deficit and levels of mitochondrial fission protein, DRP1. Moreover, scientists have studied the link between iron accumulation, its effects on mitochondria, and CBD’s activity on iron-induced mitochondrial alterations. In rats with brain iron accumulation, CBD restored succinate dehydrogenase activity, increased the levels of mitochondrial ferritin and enhanced the hydroxymethylation of the mitochondrial DNA. Considering that these effects may be correlated with bioenergetic recovery and neuroprotective properties, CBD’s activity could promote the survival of neural cells and thus could be used for the treatment of neurodegenerative diseases [68]. Due to its antioxidative profile, CBD can protect against oxidative injury, as was noticed in striatal neurons exposed to 3-nitropropionic acid, a complex II inhibitor [69]. Similarly, CBD scavenges ROS and enhances oxidative stress resistance in Alzheimer’s disease induced in *C. elegans*. The respective antioxidative effect represents a novel therapeutic strategy for Alzheimer’s disease since oxidative stress promotes the amyloid–beta aggregation [70]. Furthermore, another study indicated that CBD also has an effect on respiratory chain complexes, increasing the activity of complexes I, II, III and IV (Figure 10) [31]. Interestingly, CBD did not affect the mitochondria or the oxidative status in the case of foetal alcohol spectrum disorder induced in mice. As a possible explanation for the lack of mitochondrial targeting, the researchers proposed that protein and lipid metabolism may be involved in the studied model [71].

### 3.7. CBD and Mitochondria in Gastrointestinal Pathologies

**Figure 11:**
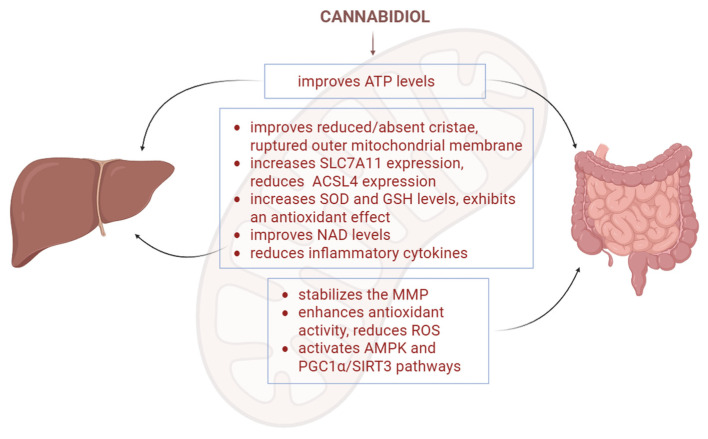
CBD’s effects on mitochondria in gastrointestinal and liver pathologies. In the liver pathology model, CBD improved ATP levels, improved reduced cristae and ruptured outer mitochondrial membrane, increased SLC7A11 and decreased ACSL4 expression, increased antioxidant enzyme levels, improved NAD levels and reduced inflammatory cytokines. In the gastrointestinal pathology models, CBD improved ATP levels, stabilised the MMP, reduced ROS and activated AMPK and PGC1a/SIRT3 pathways. SOD—superoxide dismutase; GSH—glutathione; MMP—mitochondrial membrane potential. The figure was created with the help of BioRender.com.

Ulcerative colitis (UC) is a form of inflammatory bowel disease, characterised by inflammation of the mucosa in the colon and rectum [72]. Previous research has shown an important correlation between oxidative mitochondrial function, oxidative stress, and ulcerative colitis [73]. One study reported that a formulation containing CBD protected mitochondrial functions by stabilising the mitochondrial membrane potential and increasing the ATP production in cells, and restored the mitochondrial ATP levels in colon tissue of UC model mice, thus suggesting its potential to reduce mitochondrial dysfunction and subsequent inflammation associated with ulcerative colitis (Figure 11) [30]. Furthermore, recent findings further highlight the beneficial effects of CBD on mitochondrial signalling and oxidative stress in intestinal epithelial cells. For instance, in Caco-2 cells, CBD was shown to reduce ROS levels, enhance antioxidant enzyme activity, and activate AMPK and PGC1α/SIRT3 signalling pathways, all of which contribute to improved mitochondrial function and intestinal barrier integrity [74].

### 3.8. CBD and Mitochondria in Liver Pathologies

Liver diseases represent the eleventh leading cause of death worldwide, and it is estimated that 2 million people die annually from hepatic pathologies. CBD is a promising therapeutic compound, as it has shown effects against various liver diseases [75].

Stress-induced liver injury is characterised by hepatocyte deformation and fibrosis, accompanied by inflammation and oxidative stress [76]. Huang et al. [76] used a mouse model of stress-induced liver injury to investigate CBD’s effects on this pathology, as CBD has shown promising antioxidant and anti-inflammatory effects. The mice were given 20 mg/kg of CBD three times a day. CBD treatment not only improved the affected mitochondrial morphology (reduced/absent cristae and ruptured outer mitochondrial membrane) but also increased SOD levels and SLC7A11 expression, while reducing ACSL4 expression [77]. As a result, CBD decreased the levels of transaminases and inflammatory cytokines, ultimately reducing liver damage [76]. It was also reported that CBD decreases ethanol-induced injury, restoring previously reduced ATP levels while exhibiting an antioxidant effect at the same time [77].

Another study, done by Silvestri et al., proposed that CBD increases mitochondrial activity in hepatosteatosis by elevating ATP, glutathione, and NAD levels (Figure 11) [78].

### 3.9. CBD and Mitochondria in Muscle Conditions

Excessive physical activity has a negative effect on mitochondria, causing dysfunction, decreased anabolism, fusion and fission, as well as excessive mitophagy. Si et al. studied the effects of CBD on rats that were exposed to exhaustive exercise training and the results showed that CBD improves both mitochondrial structure and function. The exact mechanism that lies behind these effects is the inhibition of excessive mitophagy, mediated indirectly through the PINK/PARKIN and BNIP3 pathway. These observations are in agreement with previous studies, indicating that CBD improves the recovery from excessive exercise [24]. Another study showed that CBD has beneficial effects in the treatment of chemotherapy-induced cachexia by preventing mitochondrial alterations, cell death, and muscle atrophy. The experiment included myotubes that were exposed to chemotherapeutic drugs (cisplatin and oxaliplatin) and CBD. CBD treatment reduced the chemotherapy-elevated expression of mitochondrial complex I marker, NDUFB8, and decreased the mitophagy marker, parkin, the latter effect being observed only in the cisplatin group (Figure 6) [79].

## 4. Dose–Response Relationship

Even with the acknowledged advantages of CBD administration, knowledge about its pharmacokinetics and metabolism remains limited [80]. Many preclinical studies on CBD and mitochondria have used concentrations that exceed the highest plasma levels recorded in humans, often surpassing clinically relevant exposures [8,81]. Furthermore, some human and animal studies administered CBD intravenously, bypassing lymphatic absorption and increasing hepatic first-pass extraction, while other oral studies often included co-administration with THC, a structurally and lipophilically similar compound that affects CBD metabolism and distribution. Most studies have focused on short-term administration, and the effects of long-term CBD intake on tissue accumulation remain poorly understood [80].

Table 2, Table 3 and Table 4 summarise studies that investigated the effects of CBD on mitochondrial function in various in vitro and in vivo models, and one clinical model, highlighting the doses used and the corresponding responses.

In cancer models, in vitro, CBD was tested at doses ranging from approximately 1 to 100 µM, and the anticancer effect was observed to be dose-dependent. Higher doses were associated with reduction in viability and apoptosis, depolarisation of the mitochondrial membrane, calcium influx, and increased ROS production, whereas lower doses caused more subtle changes in mitochondrial parameters. In one in vivo study, repeated intraperitoneal administration of 20 mg/kg three times per day for one week in BALB/c nude mice injected with MKN45 human gastric cancer cells resulted in slower tumour growth.

Besides the anticancer effect, other studies showed that CBD can have protective effects against some side effects of chemotherapeutics. In vitro, CBD showed protective effects in chemotherapy-treated cells at 5 µM for cisplatin and 12 µM for paclitaxel (PTX), with stronger effects when delivered via extracellular vesicles. In vivo, protection was observed across 2.5–10 mg/kg/day for cisplatin-induced renal dysfunction, 26 mg/kg/day for doxorubicin-induced kidney damage, 10 mg/kg for doxorubicin-induced cardiomyopathy, and 5 mg/kg in combination with PTX in neuronal tissue These findings highlight that CBD’s protective effects are dose-dependent and chemotherapy-specific.

In hepatic models, CBD showed dose-dependent effects, reducing lipid levels in HHL-5 hepatocytes (1–10 µM) and reversing ATP decline in C57Bl/6 mice with liver injury (5 mg/kg every 12 h). In cardiac models, CBD was cardioprotective in H9C2 cells (10 µM) and reduced fibrosis in vivo, with maximal effects at 1 mg/kg and higher doses less effective; in pulmonary arterial hypertension, 10 mg/kg was more effective than 20 mg/kg. In muscle and neuronal models, in vitro protection was strongest at 5 µM in HT22 neurons, while in vivo, 70 mg/kg improved muscle mitochondrial function. In intestinal Caco-2 cells, 10 µM CBD ameliorated ROS production, and in CL4176 worms, CBD delayed paralysis dose-dependently (0–100 µM). One clinical ex vivo study in rheumatoid arthritis showed stimulation at ~1 µM and decreased viability and inflammatory markers at doses above 5 µM.

Overall, the data indicate that CBD modulates mitochondrial function in a manner that depends on dose, pathology, and biological context.

## 5. Formulations for Enhancing CBD’s Mitochondrial Activity

Although CBD presents therapeutic potential, its clinical use is limited due to (1) non-selective cytotoxicity [55], and (2) low bioavailability, caused by poor solubility, easy decomposition [30] and intense hepatic metabolisation [82]. Hence, several strategies are used to overcome these challenges, including the use of nanotechnology-based strategies. The potential of nanoparticle-based delivery systems has also been extensively explored to address the poor bioavailability and off-target effects of hydrophobic compounds [84]. For example, Liu et al. synthesised conjugates between CBD and mitochondria-targeting triphenylphosphonium (MTTPP), which, when added an ether chain linker, improved mitochondrial morphology, increased ATP levels, and led to an overall decrease in cytotoxicity by reducing off-target effects [55]. MTTPP is a lipophilic cation and can, therefore, penetrate the biological membranes and target negatively charged mitochondria (negative MMP), to which it is ellectrophoretically attracted. It is often used for aimed delivery of drugs [30,85] A study focused on mitochondrial-targeted nanoparticles containing inulin and α-lipoic acid polymer and decorated with MTTPP. The respective nanoparticles were then tested against ulcerative colitis where they maintained the mitochondrial membrane potential, improved the ATP levels, and restored the mitochondrial function. As expected, the effects of the mitochondrial-targeted nanoparticles were more pronounced in comparison with free CBD [30]. Consistent findings were described by Zhang et al. who used deoxycholic acid, pullulan polysaccharide, and α-lipoic acid to incorporate CBD in nanoparticulated systems. The respective nanoparticles were synthesised in order to increase the bioavailability and stability of CBD, while enabling its accumulation at the target site. Pullulan contributed to the liver-targeting properties of the formulation due to its affinity for the hepatic tissue, while α-lipoic acid has the ability to release the drug under high glutathione concentrations, often found in cancer cells when tested in HepG2 cells, which served as a model for investigating the prevention of acute liver injury. These nanoparticles exhibited significant accumulation in the liver cells and promoted CBD internalisation. Consequently, CBD reduced the elevated oxidative damage induced by H_2_O_2_ while maintaining the MMP, indicating its potential for liver cell protection. The nanoparticles exhibited superior effects than CBD in free form [86]. Although HepG2 cells are cancer-derived, and these results might suggest a protective effect on cancer cells, studies show that ROS have a context-dependent and potentially paradoxical role in cancer: ROS are often already elevated and act as signalling molecules which promote proliferation and survival, so reducing ROS levels may interfere with cancer-promoting pathways rather than support tumour growth [87]. Given that CBD is a hydrophobic compound, Alcantara et al. formulated lipid-based nanoparticles for its incorporation, which were further tested in an inflammation-induced cell model. Compared to the CBD free form, the nanoparticles reduced ROS levels in a superior manner. The solid-lipid nanoparticles offer an increase in bioavailability, biologic activity and absorption, thus improving therapeutic efficiency [83]. Another way to increase its bioavailability is to incorporate CBD in extracellular vesicles; the vesicles originating from human umbilical cord-derived mesenchymal stem cells were tested on C57BL/6J mice that suffered from neuropathic pain. The results have shown that the vesicles reduced the mitochondrial dysfunction and alleviated thermal hypersensitivity more efficiently than free CBD. Besides targeted drug-delivery, the human umbilical cord vesicles loaded with cannabinoids might have the property of activating the AMPK pathway, leading to improved mitochondrial function and neuroprotection [82]. Nanoparticle encapsulation of CBD was also used to increase the topical bioavailability of CBD, through enhanced penetration, greater surface–volume ratio, and stabilised degradation kinetics. When tested on human skin, the formulation protected against mitochondrial DNA injury while also reducing oxidative stress [88].

Table 5 summarises the main advantages and disadvantages of different types of CBD formulations.

## 6. Future Directions

Studies have demonstrated that CBD has pharmacological inhibitory properties, as well as anti-inflammatory, antioxidant and anti-apoptotic characteristics, indicating that it could potentially be administered as a therapeutic agent in various diseases, including neurological, psychiatric, autoimmune or cardiovascular diseases. Future research is necessary to obtain additional information regarding its precise mechanism of action, efficacy and optimal use for specific medical conditions [1].

Current knowledge regarding CBD’s therapeutic effects originates primarily from preclinical and cellular models, suggesting that more in vivo and clinical studies are needed to determine dosage, metabolic profiles, interactions with other medications and time of administration of CBD, and to investigate the potential influence of genetic and physiological variation between models or species on the outcomes [2].

CBD is a highly lipophilic substance with low water solubility, which contributes to its limited bioavailability. To address this issue, researchers have focused on different delivery systems to enhance the pharmacokinetic profile, not only through conventional formulations such as patches, films, and gels, but also via CBD-loaded nanoformulations, which offer notable advantages by facilitating drug release and increasing CBD bioavailability. However, only a limited number of nanoformulations have been explored in depth and, hence, this represents an important direction for future research. Moreover, studies have concentrated predominantly on oral, sublingual or inhalation routes of administration, therefore, alternative routes, such as transdermal and rectal delivery, warrant further investigation in future studies [90].

Current studies suggest that CBD has relevant effects on mitochondrial function, which should be further investigated to determine its therapeutic potential, especially in conditions associated with mitochondrial dysfunction. The field of research is still at an early stage and meaningful comparisons between studies are difficult due to large differences in doses and routes of administration. Future studies should focus on testing the CBD effects on mitochondria at realistically achievable concentrations in humans according to pharmacokinetic data, as the concentrations used in many in vitro studies might be overestimated in the available literature [8]. In addition, future research should also include a wider range of tissue and cell types, as it was demonstrated that CBD can affect mitochondrial processes differently across distinct cell types, even within the same tissue. Regarding in vivo studies, many of them utilise the intraperitoneal injection as a route of administration, which has limitations for use in humans, while the main routes of administration in humans are oral, intravenous, and inhalation [8].

Different CBD formulations have been used to target mitochondrial function in various diseases, as reviewed in this paper. However, they all face some limitations. The topical nanoencapsulated CBD-loaded cream applied to combat UV-A-induced nuclear and mitochondrial DNA injury faced several limitations, including a small sample size, single- centre study and use of vehicle control in the analysis, indicating that further studies are necessary to address these issues, as well as to allow comparisons with standard UV-A protection, test effects in darker skin tones and evaluate potential side effects [88]. CBD-loaded extracellular vesicles from human umbilical cord mesenchymal stem cells, used to reduce the paclitaxel-induced peripheral neuropathy by modulating AMPK and mitochondrial function, require further investigations to confirm this hypothesis [82]. CBD-loaded solid lipid nanoparticles, which ameliorated the inhibition of pro-inflammatory cytokines and free radicals in an in vitro inflammation-induced cell model, need future research to investigate their in vivo efficacy, confirm the therapeutic potential and safety profile and explore the long-term stability of this formulation [83]. A liver-targeted CBD nanoparticle delivery system based on redox response for the prevention of acute liver injury, developed in a separate study, requires further investigations of its underlying mechanisms in order to increase the therapeutic use of these formulations [86].

Several studies have used Western blot analysis to investigate the molecular effects of CBD on mitochondrial functions and related pathways. In Parkinson’s disease models, Western blot analyses examined proteins such as Tyrosine hydroxylase and SIRT1, as well as signalling pathways including NF-kB and NOTCH to evaluate the effects on mitochondrial function [91]. In brain iron overload models, Western blot analyses examined proteins such as DNM1L, OPA1, synaptophysin, and caspase 3 to evaluate the effects of CBD on mitochondrial dynamics, synaptic integrity, and apoptosis [92]. In human monocytes, Western blot analyses examined cytochrome c to confirm CBD-induced mitochondrial targeting and activation of apoptosis-related pathways. The study also highlighted the role of Bcl-2 family proteins in maintaining mitochondrial integrity, as described in previous studies [93]. Therefore, investigation of Bcl-2 family proteins, as well as other mitochondria-related proteins, could be considered to fully elucidate the underlying mechanisms and optimise the therapeutic potential of CBD in diseases where its beneficial effects have been demonstrated.

Regarding mitochondrial respiration, one study has demonstrated that CBD, mainly at higher doses, increases the activity of complexes I, II, II-III and IV in the rat brain [31]. On the other hand, another study has shown that CBD exhibits a dose-dependent inhibitory effect on electron transport chain activity of brain mitochondria, with a predominant effect on I-III complexes at micromolar concentrations [94]. This finding is supported by Rupprecht et al., who reported that the combination of THC and CBD decreased mitochondrial respiration, by reducing the levels of subunits of complexes I and IV in glioma cells, although further studies are needed to understand the underlying mechanisms [44]. Based on the results regarding CBD’s effect on mitochondrial respiration, Valvassori et al. [31] reported that CBD could act as a double-edged sword, with nanomolar concentrations exhibiting a stimulatory effect and micromolar concentrations inhibiting mitochondrial respiration. For this reason, future research should compare an extended range of CBD concentrations using the same experimental conditions, with the aim of clarifying this dose-dependent response [8].

## 7. Conclusions

This study presents the effects of cannabidiol in multiple pathologies, focusing on its ability to profoundly influence mitochondrial functions and processes: from ROS synthesis, ATP production, to biogenesis, fusion and fission and calcium homeostasis. The selective cytotoxic effect of CBD with the induction of apoptosis is demonstrated in the case of cancer, while the cytoprotective effects are observed in the case of cardiovascular, pulmonary, neurological, hepatic, gastrointestinal, and muscular diseases, but also in the autoimmune disease rheumatoid arthritis. The pharmacological effects of CBD are strongly correlated with its chemical structure, hence the modification of the structure can lead to an improved activity. However, the clinical use of CBD is limited by its low bioavailability and non-selective cytotoxicity. In this regard, recent research proposes innovative formulation strategies—such as mitochondria-targeted nanoparticles, conjugation with triphenylphosphonium or incorporation into extracellular vesicles—that improve the stability, absorption and therapeutic efficacy of CBD. In conclusion, CBD represents a molecule with remarkable therapeutic potential, and its targeting to mitochondria opens new perspectives in the treatment of chronic and degenerative diseases. However, further studies are needed to fully understand the underlying molecular mechanisms and to optimise formulations in order to maximise the efficacy and safety of this promising compound.

## Figures and Tables

**Table 2:** CBD’s effects on mitochondrial function in various in vitro studies.

	In Vitro Studies				
Cell Type	Cell Line	Formulation	Dose	Effect	Reference
Glioma cells	U87	CBD → ethanol → culture media	0, 20, 30 and 50 µM	Dose-dependent decrease in viability.	[38]
			10 and 25 µM	A dose-dependent activity was observed: at the lower concentration (10 µM), there was no caspase-3, caspase-8 and caspase-9 activation, no citocrome c release, and no GSH depletion. On the contrary, the higher dose (25 µM) lead to caspase-3, caspase-8 and caspase-9 activation, citocrome c release, and GSH depletion.	
		CBD → DMSO → culture media	20, 30 μM	Dose-dependent cytotoxic activity, with an expression of autophagy biomarkers, and upregulation of endoplasmic reticulum stress markers.	[25]
		CBD isolate and extract → DMSO → ethanol/ultra-pure water	0, 0.5, 1, 2.5, 5, 7.5, 10 and 20 μM	Lower concentrations (0.5–7.5 μg/mL) did not exhibit cytotoxic effects, whereas higher concentrations (10–20 μg/mL) caused cell death. Still, a decrease in ATP production was observed at both lower and higher concentrations.	[39]
		CBD → DMSO	1, 5, 10, and 20 μM	Dose-dependent reduction in cell viability, with 1 μM being the minimal significant dose, causing an alteration of mitochondrial membrane potential, activation of apoptosis and an increase in Nrf-2 level.	[42]
	U118 MG	CBD → DMSO → culture media	20, 30 μM	Observed cytotoxic activity was dose-dependent.	[25]
	LN18	CBD → DMSO → culture media	20, 30 μM	The cytotoxic activity increased with increasing dose.	[25]
	J3TBG	CBD isolate and extract → DMSO → ethanol/ultra-pure water	0, 0.5, 1, 2.5, 5, 7.5, 10 and 20 μM	A decrease in ATP production was observed at lower and higher concentrations; however, at 7.5 μg/mL, there was still no cytotoxic activity, 10 μg/mL being the first concentration to cause cytotoxic effects, and 20 μM being the most effective dose.	[39]
	SDT3G	CBD isolate and extract → DMSO → ethanol/ultra-pure water	0, 0.5, 1, 2.5, 5, 7.5, 10 and 20 μM	Dose-dependent cytotoxic effect, starting at 10 μg/mL. Both lower and higher concentrations caused a decrease in ATP production.	[39]
	U373MG	CBD isolate and extract → DMSO → ethanol/ultra-pure water	0, 0.5, 1, 2.5, 5, 7.5, 10 and 20 μM	Cytotoxic effects were observed at 10 μg/mL and 20 μg/mL; a decrease in ATP production was also observed in lower concentrations.	[39]
Leukaemia cells	Jurkat	CBD → methanol → culture media	1, 10, 30, 60 and 100 μM	A dose-dependent activity was observed: at the lowest tested concentration (1 μM), the compound led to the proliferation of Jurkat cells; at a higher dose (10 μM), the proliferation was stopped, but the cells remained alive; at doses higher than 30 μM, cell death by apoptosis was observed. Also, a dose-dependent increase in ROS, elevation of calcium levels and decrease in mitochondrial membrane potential were detected.	[11]
		CBD → ethanol/methanol	0–100 μM	Dose-dependent cytotoxic effects; the loss of mitochondrial membrane potential was observed at 30 μM.	[39]
	CCFR-CEM	CBD → methanol → culture media	1, 10, 30, 60 and 100 μM	Dose-dependent cytotoxic effects.	[11]
		CBD → ethanol/methanol	0–100 μM	Dose-dependent cytotoxic effects.	[47]
	MOLT-3	CBD → methanol → culture media	1, 10, 30, 60 and 100 μM	Dose-dependent cytotoxic effects.	[11]
	MOLM-6	CBD → DMSO	Initial screening: 10–75 μM. Subsequent evaluations: IC50 doses (25 μM)	A dose-dependent reduction in cell viability via TRPV2 activation was observed.	[49]
	K562	CBD → methanol → culture media	1, 10, 30, 60 and 100 μM	Cytotoxic effects were observed only at higher doses (100 μM).	[11]
		CBD → DMSO	Initial screening: 10–75 μM. Subsequent evaluations: IC50 doses (KU812-15 μM, K562-20 μM, MOLM-6-25 μM)	A dose-dependent reduction in cell viability via TRPV2 activation was observed.	[49]
	Reh	CBD → methanol → culture media	1, 10, 30, 60 and 100 μM	Dose-dependent cytotoxic effects.	
	RS4	CBD → methanol → culture media	1, 10, 30, 60 and 100 μM	Dose-dependent cytotoxic effects.	[11]
	KU812	CBD → DMSO	Initial screening: 10–75 μM. Subsequent evaluations: IC50 doses (KU812-15 μM, K562-20 μM, and MOLM-6-25 μM)	A dose-dependent reduction in cell viability via TRPV2 activation was observed.	[49]
Prostate cancer cells	Non-HRPC TRAMP-C2		Dose (µM): 1, 3, 6, 10, 15, 30, 100	Cytotoxic effects were observed at doses higher than 6 µM.	[50]
	HRPC TRAMP-C2		Dose (µM): 1, 3, 6, 10, 15, 30, 100	At 6 µM, CBD caused mitochondrial fragmentation and swelling, ↑ mtROS, increased autophagy, mitochondrial fission/fusion changes, decreased maximal respiration (Complex I–IV); cytotoxic effects were observed at doses higher than 6 µM.	[50]
Gastric cancer cells	MKN45	CBD → ethanol	0–10 μM	Dose-dependent reduction in viability and apoptosis.	[26]
	MKN74	CBD → ethanol	0–10 μM	Cell viability decreased in a dose-dependent manner.	[26]
	AGS cells	CBD → ethanol	0–10 μM	Dose-dependent reduction in cell viability.	[26]
Breast cancer cells	MDA-MB-231	CBD → DMEM	0–10 μM	Dose-dependent cell death, apoptosis, autophagy, and dissipation of mitochondrial membrane potential.	[27]
	MCF-10A	CBD → DMEM	0–10 μM	CBD led to a dose-dependent cell death.	[27]
	MCF-7	CBD → DMEM	0–10 μM	CBD reduced cell viability in a dose-dependent manner	[27]
	SK-BR-3	CBD → DMEM	0–10 μM	A dose-dependent reduction in cell viability was observed.	[27]
	ZR-75-1	CBD → DMEM	0–10 μM	Observed cytotoxic effects was dose-dependent.	[27]
Lymphoma cells	Canine B-cell lymphoma 1771	CBD → ethanol	0.5–50.0 μM	At lower doses (0.5–1 μM) no cytotoxic effects were observed; doses higher than 25 μM led to a significant reduction in cell viability. Also, a dose-dependent increase in ROS generation, nitrite content and NADH, and decrease in glutathione were mentioned.	[54]
	CLBL-1	CBD → ethanol	0.5–50.0 μM	Concentrations of 0.5 and 1 μM) caused a rise in cell viability; doses of 25 μM and 50 μM led to a significant reduction in cell viability.	[54]
	T-cell lymphoma CL-1	CBD → ethanol	0.5–50.0 μM	At lower doses (0.5–1 μM) no cytotoxic effects were observed; doses higher than 25 μM led to a significant reduction in cell viability.	[54]
	Human Burkitt’s lymphoma cell line Ramos	CBD → ethanol	0.5–50.0 μM	At lower doses (0.5–1 μM), CBD exhibited a significant stimulatory effect; on the contrary, doses higher than 25 μM caused a reduction in cell viability.	[54]
Cardiomiocytes	H9C2	Not mentioned	10 μM	A single concentration was used, which showed that CBD has cardioprotective effects	[65]
Neuronal cells	HT22 under ischemic conditions	Not mentioned	1, 2.5, 5, or 10 μM	The most effective protection against oxygen–glucose-deprivation/reperfusion occurred at 5 μM	[5]
	Primary rat DRG neurons		12 µM free CBD or 12 µM CBD-extracellular vesicles (Evs) (after 3 µM paclitaxel)	At 12 µM, free CBD restored mitochondrial function by increasing MMP, ATP levels, and p-AMPK expression; at the same concentration, CBD-EVs showed similar but stronger effects	[82]
Colorectal cancer cells	Caco-2	CBD hemp oil/CBD → DMSO	10 μM	CBD can ameliorate ROS production at 10 μM.	[74]
Liver cells	HHL-5 induced model of hepatosteatosis	Not mentioned	1–10 μM	Dose-dependent decrease in lipid levels.	[78]
Muscle cells	C2C12	Not mentioned	5 μM	A single tested concentration resulted in the prevention of cisplatin-induced atrophy and apoptosis, reducing NDUFB8 and parkin upregulation.	[79]
Chondrocytes	SW 1353	CBD-loaded solid lipid nanoparticles	0.125 to 1.0 μg/mL	Cytotoxic effects were observed only at the highest tested concentration, 1.0 μg/mL. A significant reduction in ROS occurred at 0.5 μg/mL, while IL-6 decreased in a dose-dependent manner.	[83]
Macrophages	RAW 264.7	CBD-loaded solid lipid nanoparticles	0.125 to 1.0 μg/mL	Cytotoxic effects were observed only at the highest tested concentration, 1.0 μg/mL. At an intermediate dose (0.5 μg/mL), a significant ROS inhibition was observed.	[83]
	RAW264.7	Not mentioned	0.78125, 0.1, 0.2, 0.4, 0.8, 1.5625, 1.6, 3.125, 3.2, 6.25, 12.5, 25, 50, 100, 200 μg/μM	At lower doses (≤0.8 μg/mL), CBD did not exhibit cytotoxic activity; however, at doses higher than 1.5625 μg/mL, cell viability was significantly reduced (10%).	[30]

Note: ↑ indicates increase.

**Table 3:** CBD’s effects on mitochondrial function in various in vivo studies.

		In Vivo Studies			
Animal	Model	Specifics	Dose	Effect	Reference
Worms	CL4176	-	0–400 μM	At lower doses (0–100 μM), a dose-dependent delay of paralysis was observed. At the highest tested concentration (400 μM), CBD showed inferior activity than at 100 μM, probably by causing stress.	[70]
	*C. elegans*	-	100 μM	At 100 μM, CBD successfully decreased ROS.	[70]
Mice	C57BL/6	Mice with induced liver injury	5 mg/kg, every 12 h for 5 days	Single-tested concentration; treatment-reversed ethanol-induced decline in ATP and reduced triglyceride levels.	[77]
		Male mice with induced pulmonary arterial hypertension	10 mg/kg and 20 mg/kg, e.g., for 13 days	After administering a 10 mg/kg dose, CBD successfully ameliorated pulmonary arterial hypertension; moreover, better efficacy was observed for 10 mg/kg than for 20 mg/kg.	[28]
		Male mice with cisplatin-induced renal dysfunction	2.5 to 10 mg/kg, every day, for 3 days, intraperitoneally	Dose-dependent amelioration of cisplatin-induced renal dysfunction.	[60]
		Male mice with doxorubicin-induced cardiomyopathy	10 mg/kg, once a day, for 5 days, intraperitoneally	A single concentration was tested and it showed that CBD protects against doxorubicin-induced cardiomyopathy.	[64]
		Male mice with induced heart failure	0.1, 1, or 10 mg/kg every third day, for 4 weeks. Afterwards, 1mg/kg, every third day, for 4 weeks	A dose-dependent reduction in cardiac fibrosis was observed; 1 mg/kg was the most effective concentration and the therapeutic effect appeared to plateau; alternatively, the highest tested concentration (10 mg/kg) did not lead to benefits compared to lower doses.	[29]
		Perfluorooctanesulfonic acid (PFOS)-induced heart injury	10 mg/kg	Single concentration was tested and it showed that CBD can improve the antioxidant capacity and PFOS-induced mitochondrial dynamics imbalance and energy metabolism.	[65]
		Female, treated with paclitaxel	5 mg/kg CBD (i.p.), ±paclitaxel, extracellular vesicles, WAY100135, or rimonabant, twice weekly for 6 weeks after the last paclitaxel dose, or daily 3 h after receptor blockers	Restored ATP and NAD^+^ levels; increased MMP; upregulated p-AMPK, SIRT1, SIRT3, Parkin, SOD2, Catalase, NRF1; mitoprotective effects stronger with CBD-extracellular vesicles; all mitochondrial effects observed only with these formulations after paclitaxel treatment.	[82]
	BALB/c nude mice	Mice injected with MKN45 cells	20 mg/kg/3 times per day/one week injected intraperitoneally	Single concentration was tested that led to a slower tumour growth.	[26]
Rats	Sprague– Dawley rats	Male rats exposed to exhaustive exercise training	50 mg/kg, 60 mg/kg, and 70 mg/kg, intraperitoneal injection, once a day, for 9 days	The best results regarding improvement of muscle mitochondrial dysfunction were observed at the highest dose (70 mg/kg).	[24]
		Male rats that were administered doxorubicin	daily dose of 26 mg/kg, orally, for 2 weeks	One concentration was tested and it led to a protective effect against doxorubicin-induced kidney abnormalities.	[62]
	Wistar rats	Male rats with or without induced pulmonary trauma	5 mg/kg, single dose, intraperitoneally	One concentration was tested and it showed that CBD reduces lung damage. A use of higher doses for future research was proposed in order to increase the antioxidative effects.	[67]

**Table 4:** CBD’s effects on mitochondrial function in clinical studies.

		Clinical Studies	
Patients	Dose	Effect	Reference
patients with rheumatoid arthritis → synovial tissue	0.5–20.0 μM	At a lower dose (1 µM), CBD exhibited a stimulatory effect; at doses higher than 5 μM a decrease in cell viability, IL-6, IL-8, and MMP-3 levels and increase in intracellular calcium was observed.	[58]

**Table 5:** Comparison between free CBD and nanoformulations: main advantages and disadvantages (Based on Paczkowska-Walendowska et al. [89] and Zhang et al. [30]).

Category	Advantages	Disadvantages
Free CBD	Oral bioavailability can increase up to four times when taken with an FDA/EMA-approved high-fat meal.	Very low oral bioavailability due to negligible water solubility and significant first-pass metabolism (~75% eliminated).
	Non-psychoactive phytocannabinoid; well tolerated; may counteract THC intoxication.	Prone to light-induced degradation, limiting shelf life.
	Highly lipophilic, enabling integration into lipid environments.	Non-specific distribution; absorption/degradation in the upper GI tract before reaching the colon.
	Simple production processes.	Poor skin penetration in standard transdermal solutions (e.g., PEG 400).
	Extensive clinical data available.	Slow colorectal permeation after rectal administration.
		Vapourisation/sublingual administration may cause throat irritation; reduced efficiency if sublingual oils are swallowed.
CBD Nanoformulations	Oral (SNEDDS/nanoemulsions): markedly increased solubility and bioavailability.	Physical instability (CBD leakage, vesicle fusion, and lipid oxidation during storage).
	Targeted oral nanoparticles (polymeric/inulin): enhanced GI stability; colon-specific release triggered by microbiota or redox conditions (GSH-responsive).	High surfactant concentrations (e.g., in SNEDDS) may cause GI irritation or cytotoxicity.
	Transdermal systems (liposomes/ethosomes): 2–4× higher skin permeability compared with standard solutions.	Advanced nanosystems (e.g., pro-nanolipospheres) may inhibit metabolic enzymes, potentially causing accumulation of co-administered drugs.
	Rectal delivery (transferosomes): ~70% increase in bioavailability; deep tissue penetration; ~95% release within 7 h.	Complex and costly scale-up; lack of standardised global toxicological guidelines.
	Intramuscular nanosuspensions: higher maximum concentration than oral oils.	Possible “burst effect” (~35% released within the first hour) in some polymeric systems.
	Lymphatic delivery (oleosomes): 26.5× higher CBD levels in lymph fluid; bypasses hepatic first-pass metabolism.	Limited long-term clinical studies for some nanosystems

## Data Availability

No new data were created or analysed in this study. Data sharing is not applicable to this article.

## Abbreviations

The following abbreviations are used in this manuscript:

ATP	Adenosine triphosphate
CBD	Cannabidiol
CB1	Cannabinoid receptor 1
CB2	Cannabinoid receptor 2
FDA	Food and drug administration
GBM	Glioblastoma multiform
GSH	Glutathione
HKII	Hexokinase II
HRPC	Hormone-refractory prostate cancer
MAO	Monoamino oxidase
MCU	Mitochondrial calcium uniporter
MDA	Malondialdehyde
MMP	Mitochondrial membrane potential
mPTP	Mitochondrial permeability transition pore
MTTPP	Mitochondria-targeting triphenylphosphonium
NCX	Na/Ca exchanger
NHL	Non-Hodgkin lymphoma
PAH	Pulmonary arterial hypertension
RANKL	Receptor activator of NF-κB ligand
ROS	Reactive oxigen species
SOD	Superoxide dismutase
T-ALL	Acute lymphoblastic leukaemia
THC	Tetrahydrocannabinol
TNF	Tumour necrosis factor
TRPA1	Transient receptor potential ankyrin
TRPV	Vaniloid receptors-transient receptor potential cation channel
UK	United Kingdom
VDAC	Voltage-dependent anion channel
XIAP	X-linked inhibitor of apoptosis protein
